# Examining the association between personality traits and university faculty: a web-survey among Italian students

**DOI:** 10.1192/j.eurpsy.2022.476

**Published:** 2022-09-01

**Authors:** A. Ciancio, L. Fusar-Poli, A. Gabbiadini, G. Saitta, M.S. Signorelli, E. Aguglia

**Affiliations:** University of Catania, Department Of Clinical And Experimental Medicine, Psychiatry Unit, Catania, Italy

**Keywords:** big five personality, faculty, UNIVERSITY, career

## Abstract

**Introduction:**

The influence of personality on field of study choice is comparable to that of cognitive skills. Additionally, personality traits seem linked with academic motivation, and engagement. Choosing the most suitable career is also related to students’ personal well-being and work success.

**Objectives:**

To explore how personality traits are associated with the choice of university courses among Italian students.

**Methods:**

A web-survey was spread on social networks between March and June 2020 through Google Forms. Eligibility criteria for inclusion were: 1) Being a university student between 18 and 35 years of age; 2) Attending a course in an Italian university; 3) Good comprehension of Italian language. On-line informed consent, socio-demographic, and career data were collected during the survey. Personality traits were assessed using the Big Five Inventory (BFI). We computed multinomial linear regressions to calculate potential associations between personality traits and university courses.

**Results:**

Lower Conscientiousness, higher Neuroticism, and higher Openness to experience are associated with the attendance of Humanities compared with students of Health faculties. Higher Neuroticism traits are associated with the attendance of a scientific course compared with Health faculties. High Conscientiousness is significantly associated with the attendance of Law-related courses compared with Health courses. Non significant differences were detected in the other domains according to the big five personality model.

**Conclusions:**

Our results suggest interesting associations between personality traits and educational choices. Future research may investigate this relationship in high-school students to implement appropriate strategies for better addressing students’ educational needs and career outcomes.

**Disclosure:**

No significant relationships.

